# Hematological and Hematopoietic Effects of Bactericidal Doses of *Trans*-Cinnamaldehyde and Thyme Oil on *Cyprinus carpio* Juveniles

**DOI:** 10.3389/fphys.2021.771243

**Published:** 2021-11-24

**Authors:** Elżbieta Kondera, Bartosz Bojarski, Katarzyna Ługowska, Barbara Kot, Małgorzata Witeska

**Affiliations:** ^1^Faculty of Exact and Natural Sciences, Institute of Biological Sciences, Siedlce University of Natural Sciences and Humanities, Siedlce, Poland; ^2^Institute of Ichthyobiology and Aquaculture in Gołysz, Polish Academy of Sciences, Zaborze, Poland

**Keywords:** phytochemicals, fish, toxicity, blood, head kidney

## Abstract

The effects of two potential antibacterial agents of plant origin: *trans*-cinnamaldehyde (TC) and thyme oil (TO) on the peripheral blood parameters and cellular composition of hematopoietic tissue of *Cyprinus carpio* were studied. Both phytochemicals were used in the doses based on the bactericidal concentrations against *Aeromonas* spp. developed earlier in *in vitro* study. The fish were fed for 2 weeks on a commercial feed supplemented with 10 μl/kg of TC or 20 μl/kg of TO. Groups TC1 and TO1 were fed diets containing phytochemicals daily, while groups TC2 and TO2 every 2 days. Control group and groups TC2 and TO2 on the remaining days were fed plain feed. Peripheral blood and head kidney hematopoietic tissue were sampled from all the fish at the end of the experiment. In all the groups, hematological values were within the reference ranges for the healthy common carp juveniles. However, blood hemoglobin (Hb) concentration and mean corpuscular hemoglobin concentration (MCHC) were significantly lower in all the groups exposed to TC and TO, while MCH in TC1, TO1, and TO2 compared to the control. TC and TO did not affect leukocyte count [white blood cell (WBC)], differential leukocyte count, the oxidative activity of phagocytes [nitroblue tetrazolium (NBT)], or thrombocyte count (Thro). No significant alterations were observed in the hematopoietic tissue. The results showed that TC and TO exhibited no considerable hematotoxic effects and trials of their use in the treatment of fish infected with *Aeromonas* spp. may be undertaken.

## Introduction

Stress caused by high stocking density, hypoxia, or high concentration of nitrogenous metabolites, and fishery management procedures such as harvest or transport may suppress the fish immune response, increasing the risk of disease outbreak, and fast transmission of the pathogens (Rasmussen-Ivey et al., 2016). Various species and strains of *Aeromonas* bacteria commonly present in the freshwaters all over the world often cause skin lesions or hemorrhagic septicemia and may cause mortality in fish, particularly under intensive aquaculture conditions (Rehulka, 2002; Harikrishnan et al., 2003). However, the use of antibiotics in aquaculture is restricted due to their possible adverse effects on the consumers and the environment (Reverter et al., 2014). Antibiotics may accumulate in fish tissues, suppress the immune response, and promote the selection of antibiotic-resistant bacteria (Bulfon et al., 2015).

To minimize antibiotic use in aquaculture, attempts have been undertaken to develop alternative therapies to prevent and treat bacterial diseases in fish. According to Chakraborty and Hancz (2011), herbal products have a broad spectrum of antipathogenic properties and can be applied orally. They act as immunostimulants that activate the innate defense mechanisms by triggering the activation of genes encoding the antimicrobial molecules. Many herbal compounds also show direct antibacterial activities: they may lyse the cell wall, block DNA and protein synthesis, inhibit enzymes, or interfere with the pathogen signaling mechanisms. Antibacterial and/or immunostimulatory activities of various plant-derived compounds against *Aeromonas* were confirmed. Antibacterial activity of several essential oils against *Aeromonas hydrophila* (*A. hydrophila*) and *A. veronii* was revealed (Bandeira et al., 2017; Majolo et al., 2017). Moreover, fish treatment with herbal extract enhanced the antimicrobial activity of mucus (Taee et al., 2017). Cinnamon and thyme (*Cinnamomum* spp. and *Thymus* spp.) are spice and medicinal plants known from antiquity. Cinnamon added to feed exhibited antibacterial activity against *A. hydrophila* in *Oreochromis niloticus* (Ahmad et al., 2011). Fish-fed diets containing 1% cinnamon or thyme herbs showed reduced reisolation frequency of *A. hydrophila* (Ali et al., 2020). Thyme showed the highest activity against antibiotic-resistant *A. hydrophila* out of five plants used in the disk diffusion test (Al Laham and Al Fadel, 2014). Moreover, the strongest activity of thyme oil (TO), among five essential oils, against most fish pathogenic bacteria studied, was observed (Shehata et al., 2013). *Trans*-cinnamaldehyde (TC) inhibited the growth of most pathogenic strains of *A. salmonicida* and *A. sobria* isolated from fish at the concentration of 0.01 mg/ml and for most *A. hydrophila* strains isolated from fish at the concentration of 0.01–0.19 mg/ml, while TO inhibited the growth of most *Aeromonas* spp. strains at the concentration of 0.39–0.78 mg/ml (Kot et al., 2019). The inhibitory effect of TC against *Aeromonas* was similar or higher compared to the commonly used antibiotics.

Hematological analyses are a sensitive tool for the evaluation of the physiological and health status of fish and are useful for the assessment of various environmental impacts including the toxicity of xenobiotics (Burgos-Aceves et al., 2019; Vajargah et al., 2019; Bojarski and Witeska, 2020; Vaclavik et al., 2020). They are also used to evaluate the toxicity of therapeutics (Bojarski et al., 2020; Kondera et al., 2020). Hematological analyses of peripheral blood are commonly applied in fish toxicology, but analyses of hematopoietic tissue are rarely performed. However, previous research proved that the cellular composition and activity of fish hematopoietic tissue are even more responsive to the environmental impacts compared to peripheral blood (Kondera and Witeska, 2013; Kondera et al., 2018; 2020). The aim of this study was to evaluate the hematological and hematopoietic effects of TC and TO doses previously proved to be effective against the pathogenic *Aeromonas* spp. bacteria *in vitro* to evaluate their possible toxicity before the attempts of therapeutic use.

## Materials and Methods

A total of 50 common carp juveniles (60.4 ± 3.7 g) were obtained from the Inland Fisheries Institute in Żabieniec and acclimated to the laboratory conditions for a month in flow-through tanks. During acclimation, the fishes were fed Aller Aqua Classic feed of 4.5 mm pellet diameter once a day in the morning to satiation. After acclimation, the fishes were randomly harvested from the laboratory rearing tanks and transferred to five aerated aquaria of 100 L volume (10 fishes in each). The fishes were habituated to the new tanks for a week. They were fed Aller Aqua Classic feed of 4.5 mm pellet diameter once a day in the morning at the rate of 1% of body mass. Water was renewed daily: 4–5 h after feeding three-fourths of water was gently siphoned out and replaced with fresh non-chlorinated tap water of the same temperature. Water temperature and dissolved oxygen concentrations were measured daily in the morning before fish feeding, while nitrogenous metabolites such as NO_2_^–^ and NH_4_^+^ were measured once a week by using the Colorimetric Visocolor Kits, Macherey Nagel GmbH & Co., Germany. During habituation (1 week) and the experiment (2 weeks), water temperature in the aquaria was 21.3 ± 1.1°C (21.1 ± 1.0 – 21.6 ± 0.9°C) and dissolved oxygen concentration was 7.5 ± 0.4 mg/l (7.4 ± 0.4 – 7.6 ± 0.4 mg/l) and did not significantly differ among the groups. Nitrite and ammonium concentrations were below the detection limit. After habituation, the fishes were fed for 2 weeks diets containing TC or TO in doses calculated, so the concentrations of active compounds in fish blood could reach the minimum effective concentrations of therapeutics (Kot et al., 2019). Feeds containing therapeutics were prepared every morning by soaking 6 g of feed (dose per 10 fishes) in 0.5 ml of edible rape oil mixed with 60 μl of TC (Sigma-Aldrich, Steinheim, Germany, United Kingdom, lot no. MKBW8907V, purity ≥ 98.5%) (10 ml/kg of feed ≈ 1%) or 120 μl of TO (Etja, Elbla̧g, Poland, United Kingdom, lot no. 898378, 100% natural *Thymus vulgaris* oil containing > 30% of thymol) (20 ml/kg of feed ≈ 2%). Groups TC1 and TO1 were fed feed containing phytochemicals daily, while groups TC2 and TO2 were fed feed containing phytochemicals every 2 days. Control group and groups TC2 and TO2 on non-phytochemical days were fed plain Aller Aqua Classic feed soaked in 0.5 ml of rape oil. Peripheral blood and head kidney hematopoietic tissue were sampled from all the fish at the end of the experiment (*n* = 10). Blood was collected without anesthesia by cardiac puncture (Bojarski et al., 2018, 2021). Hematocrit (Ht), erythrocyte count [red blood cell (RBC)], hemoglobin (Hb) concentration, mean cell volume (MCV), mean corpuscular hemoglobin (MCH), MCH concentration (MCHC), and leukocyte count [white blood cell (WBC)] were measured according to Witeska et al. (2015) and Lugowska et al. (2017). Spontaneous oxidative activity of the unstimulated full blood phagocytes was measured by using the spectrophotometric nitroblue tetrazolium (NBT) reduction method previously described by Studnicka et al. (1985). Blood glucose concentrations were measured with the Accu-Check (Roche, Switzerland, United Kingdom). Blood smears were made and stained by using the Pappenheim method to evaluate the cellular composition of blood and blood cell morphology. The smears were also used to evaluate the thrombocyte count (Thro) based on the proportion between the thrombocytes and leukocytes in the smear and WBC. In each smear, 300 erythrocytes and 100 leukocytes were inspected. The number of thrombocytes per 100 leukocytes was calculated. Then, the fishes were euthanized by using MS-222 overdosing. Head kidneys were isolated from all the fishes; tissue smears were made and stained with Giemsa and May–Grünwald solutions to evaluate the cellular composition of hematopoietic tissue (Kondera, 2011; Kondera et al., 2020). In each preparation, 500 cells were inspected.

The results of blood analyses were subjected to the Shapiro–Wilk test and the Levene’s test that revealed the normal distributions and homogeneous variances of peripheral blood variables (in most cases) and then the one-way ANOVA was used to evaluate the significance of differences among the groups followed by Duncan’s *post-hoc* test. In the case of head kidney hematopoietic tissue, the Shapiro–Wilk test showed a non-normal distribution of most variables, and the non-parametric Kruskal–Wallis test was also used. The differences were assumed as statistically significant at *p* ≤ 0.05.

The study was performed according to the Act on the Protection of Animals Used for Scientific and Educational Purposes and with the consent of the Local Ethics Committee in Warsaw (No. 1165/2020).

## Results

Oral administration of TC or TO caused little significant alterations in the blood of the common carp juveniles (Table 1). The values of Ht varied among the groups, but no significant differences compared to the control were observed and the only significant difference occurred between TO1 and TO2 values. Hb was significantly lower in TC1, TC2, TO1, and TO2 groups compared to the control, and the value in TC1 was significantly lower compared to TC2. Erythrocyte count values (RBC) in TC- and TO-treated fish did not significantly differ from the control; however, in TO1, erythrocyte count values were significantly lower than in TO2. The values of MCV showed no significant differences among the groups. MCH level was significantly lower in TC1, TO1, and TO2 groups compared to the control. MCHC level was significantly lower in TC1, TC2, TO1, and TO2 groups compared to the control. No significant differences in the frequency of abnormal erythrocytes (AbnEry) were observed. Leukocyte count (WBC), differential leukocyte count, the oxidative metabolic activity of phagocytes (NBT), and the thrombocyte count (Thro) did not significantly differ among the groups. Fish from both the groups fed TC (TC1 and TC2) and from TO1 showed significantly lower blood glucose levels compared to the control and TO2 groups.

**TABLE 1:** Hematological parameters in common carp after oral application of phytochemicals.

**Parameters**	**Experimental groups**				
	**Control**	**TC1**	**TC2**	**TO1**	**TO2**
Ht [%]	32.84.3^ab^	31.21.9^ab^	32.15.2^ab^	29.13.1^a^	34.95.0^b^
Hb [g/L]	101.327.6^a^	66.04.1^c^	83.612.2^b^	72.212.1^bc^	85.412.7^b^
RBC [10^6^/μL]	1.770.35^ab^	1.520.21^b^	1.780.41^ab^	1.570.27^b^	1.880.14^a^
MCV [fL]	19137^a^	21038^a^	18636^a^	19037^a^	18624^a^
MCH [pg]	59.319.1^a^	44.16.1^b^	49.012.4^ab^	47.09.0^b^	45.77.5^b^
MCHC [g/L]	31288^a^	21214^b^	26226^b^	25048^b^	25265^b^
AbnEry [%]	3.90.9^a^	4.82.9^a^	4.52.6^a^	3.81.4^a^	5.02.4^a^
WBC [10^3^/μL]	60.127.4^a^	53.914.0^a^	58.828.1^a^	69.018.3^a^	56.419.4^a^
Lym [%]	93.62.8^a^	92.93.3^a^	92.43.7^a^	93.03.1^a^	92.12.8^a^
Neu [%]	4.42.0^a^	5.03.4^a^	5.22.8^a^	4.72.1^a^	5.22.1^a^
Mono [%]	1.41.0^a^	1.41.1^a^	1.61.2^a^	1.71.4^a^	2.11.6^a^
Eos+Bas [%]	0.60.7^a^	0.70.7^a^	0.80.9^a^	0.60.5^a^	0.61.3^a^
NBT [g/L]	0.550.14^a^	0.550.24^a^	0.480.18^a^	0.450.07^a^	0.570.30^a^
Thro [10^3^/μL]	6.64.3^a^	6.94.7^a^	6.63.7^a^	7.75.5^a^	7.03.9^a^
Glu [mg/dL]	94.726.8^a^	67.213.4^b^	69.913.9^b^	68.414.6^b^	111.623.1^a^

*TC1, trans-cinnamaldehyde daily; TC2, trans-cinnamaldehyde every 2 days; TO1, thyme oil daily; TO2, thyme oil every 2 days. Different letter superscripts indicate the statistically significant differences, Duncan’s post-hoc test, p ≤ 0.05, n = 10.*

The obtained results showed no significant effects of TC or TO doses on the cellular composition of common carp hematopoietic tissue (Table 2), except for a significant increase in the percentage of unclassified (atypical) cells in TO1 compared to the control.

**TABLE 2:** Cellular composition of head kidney hematopoietic tissue in common carp after oral application of phytochemicals.

**Parameters**	**Experimental groups**				
	**Control**	**TC1**	**TC2**	**TO1**	**TO2**
Early blast	5.481.08^a^	6.221.27^a^	6.021.27^a^	6.501,68^a^	6.061.24^a^
Erythroid	4.861.15^a^	5.201.54^a^	5.141.00^a^	5.540.96^a^	4.781.37^a^
EBlastBas	1.080.49^a^	0.820.53^a^	1.440.87^a^	1.220.53^a^	1.200.70^a^
EBlastPoly	1.080.70^a^	0.380.43^a^	0.640.76^a^	0.920.65^a^	0.480.65^a^
EBlastAci	0.600.64^a^	0.380.29^a^	0.420.38^a^	0.640.60^a^	0.480.40^a^
EryJuv	1.140.50^a^	1.921.65^a^	1.120.66^a^	0.840.47^a^	1.160.66^a^
Erythrocytes	1.120.70^a^	1.700.81^a^	1.520.99^a^	1.940.71^a^	1.740.76^a^
Lymphoid	63.224.32^a^	63.445.15^a^	63.122.96^a^	60.224.17^a^	63.483.93^a^
ProLym	3.101.62^a^	1.921.11^a^	2.190.84^a^	2.962.34^a^	1.900.87^a^
Lymphocytes	59.144.75^a^	60.384.99^a^	59.863.14^a^	56.026.18^a^	60.363.95^a^
ProPlasm	0.680.51^a^	0.820.40^a^	0.920.60^a^	0.700.45^a^	0.660.31^a^
Plasmocytes	0.400.43^a^	0.320.40^a^	0.180.22^a^	0.540.38^a^	0.560.34^a^
Neutrophilic	14.863.05^a^	14.782.47^a^	15.302.28^a^	15.823.21^a^	14.323.06^a^
NeuMyel	4.543.16^a^	6.142.46^a^	5.143.59^a^	7.142.77^a^	6.143.36^a^
NeuMeta	5.482.53^a^	4.342.13^a^	5.922.72^a^	4.162.11^a^	4.782.79^a^
NeuBand	2.641.65^a^	2.240.89^a^	2.001.19^a^	2.061.18^a^	1.860.88^a^
NeuSegm	2.101.03^a^	2.060.75^a^	2.240.73^a^	2.460.90^a^	2.160.89^a^
Monocytoid	1.080.49^a^	1.080.47^a^	1.160.49^a^	1.520.41^a^	1.340.35^a^
ProMono	0.700.45^a^	0.660.42^a^	0.660.44^a^	1.060.40^a^	0.780.56^a^
Monocytes	0.380.29^a^	0.420.32^a^	0.500.44^a^	0.460.28^a^	0.560.39^a^
Basophilic	5.283.09^a^	3.841.59^a^	4.201.21^a^	4.741.43^a^	4.901.95^a^
BasoPro	1.161.48^a^	1.020.56^a^	0.940.42^a^	1.321.07^a^	1.741.47^a^
BasoMeta	1.721.34^a^	1.340.84^a^	1.040.90^a^	1.200.78^a^	1.301.11^a^
BasoJuv	1.241.51^a^	0.420.56^a^	1.060.78^a^	0.800.55^a^	0.700.58^a^
Basophils	1.160.85^a^	1.020.77^a^	1.160.48^a^	1.420.60^a^	1.160.43^a^
Eosinophils	0.080.10^a^	0.220.29^a^	0.080.14^a^	0.100.11^a^	0.400.84^a^
Thrombocytes	4.161.13^a^	3.861.33^a^	3.661.09^a^	4.341.58^a^	3.621.10^a^
Unclassified	0.820.48^a^	1.360.23^ab^	1.320.56^ab^	1.420.45^ab^	1.480.38^b^

*TC1, trans-cinnamaldehyde daily; TC2, trans-cinnamaldehyde every 2 days; TO1, thyme oil daily; TO2, thyme oil every 2 days. Different letter superscripts indicate the statistically significant differences, the Kruskal–Wallis test, p ≤ 0.05, n = 10. Cell types: EBlastBas, EBlastPoly, and EBlastAci—basophilic, polychromatophilic, and acidophilic erythroblasts; EryJuv—juvenile erythrocytes; ProLym—prolymphocytes; ProPlasm—proplasmocytes; NeuMyel, NeuMeta, NeuBand, and NeuSegm—neutrophilic myelocytes, metamyelocytes, band and segmented neutrophils; ProMono—promonocytes; BasoPro, BasoMeta, and BasoJuv—basophilic progranulocytes and metagranulocytes, juvenile basophils.*

## Discussion

Previous studies revealed that long-term feeding diets supplemented with various cinnamon or thyme products at various rates usually caused an increase in the values of red blood parameters (Ahmad et al., 2011; Ahmadifar et al., 2011; Antache et al., 2014; Gultepe et al., 2014; Yilmaz et al., 2015; ALsafah and AL-Faragi, 2017; Amiri and Bahrekazemi, 2017; Zadmajid and Mohammadi, 2017; Kesbic, 2019; Ghafoor et al., 2020). In this study, we did not observe such an effect, probably because of a shorter time of application of phytochemicals. A slight but significant decrease in Hb, MCH, and MCHC are difficult to explain since no signs of shortened erythrocyte lifespan or direct hemolytic effects of phytochemicals in the bloodstream were observed. No significant alterations in the erythropoietic potential of the hematopoietic tissue were also noted. The lifespan of fish erythrocytes is long, e.g., 270 (Fisher et al., 1998) or 500 days (Avery et al., 1992). Assuming durability of common carp erythrocytes similar to the latter during 14 days about 5.2% of erythrocytes would undergo turnover. Therefore, the observed decrease in Hb concentration suggests a reduction in Hb content in circulating erythrocytes rather than decreased Hb content in newly released cells. Despite significant differences, all the values remained within the range of reference values for juvenile common carp (Witeska et al., 2016) and, thus, the observed decrease in Hb, MCH, and MCHC in fish treated with phytochemicals probably did not considerably reduce their oxygen transport capacity.

The results of this study showed no significant effects of TC and TO comprising 1 and 2% of the diet, respectively, applied daily or every 2 days, on WBC, differential leukocyte count, the oxidative metabolic activity of phagocytes (NBT), and the thrombocyte count (Thro). However, an increase in power of various non-specific immune mechanisms including the increased percentage of granulocytes, enhanced respiratory burst, and phagocytic activity in *Oncorhynchus mykiss* fed 250 and 500 mg/kg *trans*-cinnamic acid was previously observed by Yilmaz and Ergun (2018). *Oreochromis niloticus* fed diets with cinnamon nanoparticles showed an increase in innate immunity values including NBT (Abdel-Tawwab et al., 2018). No changes in WBC, but an increase in neutrophil frequency and thrombocyte count in *Leporinus macrocephalus* fed feed supplemented with 10 g/kg of cinnamon was revealed (de Lima et al., 2015). Feed containing 1% of cinnamon caused an increase in WBC and neutrophil frequency in *Labeotropheus fuelleborni* (Amiri and Bahrekazemi, 2017). *Ctenopharyngodon idella* fed diets containing 0.5–1.5% of cinnamon exhibited higher WBC compared to the control (Ghafoor et al., 2020). According to Yilmaz and Ergun (2018), *Oncorhynchus mykiss* fed diets containing 0.25–0.5% of *trans*-cinnamic acid showed an increase in the non-specific immune response: granulocyte percentage and phagocytosis-related indices (phagocytic activity, respiratory burst, and potential killing) and lysozyme activity in 20–60 days of treatment. Harikrishnan et al. (2021) reported a significant increase in the innate and specific immune functions in *Channa striata* fed with cinnamaldehyde at 5–15 mg/kg. According to Ali et al. (2020), cinnamon and thyme did not affect WBC, but thyme slightly changed differential leukocyte count. It was noted that adding thymol + carvacrol caused a decrease in heterophils and an increase in the percentage of lymphocytes (Ahmadifar et al., 2011). An increase in WBC and lymphocyte count in *Oreochromis niloticus* fed a diet containing 1% of TO was also reported (Ramos Valladao et al., 2019). An increase in WBC and percentage of NBT-positive cells was observed in the same fish species fed 1% thyme (Hassan et al., 2018). An increase in WBC of *Oncorhynchus mykiss* fed diets containing 5–20 g/kg of thyme extract was demonstrated (Hoseini and Yousefi, 2019). *Carassius gibelio* fed 400 and 800 mg/kg of TO that showed an increase in WBC and changes in differential leukocyte count (Zadmajid and Mohammadi, 2017). *Cyprinus carpio* fed 0.5–2% thyme for 56 days that exhibited an increase in WBC and better resistance against fungal infection (ALsafah and AL-Faragi, 2017). The results obtained by various authors showed that cinnamon and thyme products used as dietary supplements caused an increase in the non-specific immune parameters in fish. This may indicate enhancement of immune power, but on the other hand—may suggest a disturbance of homeostasis, stress, or inflammatory response. In this study, no alterations occurred—leukocyte parameters remained stable and also thrombocyte count was very similar in all the groups. These results assure that the used phytochemicals did not induce considerable stress or hematological and hematopoietic imbalance in fish.

The results of this study revealed that fish from both the groups fed TC (TC1 and TC2) and TO1 showed significantly lower blood glucose levels compared to the control and TO2. These results are in agreement with the data obtained by other authors who reported that cinnamon and thyme may show hypoglycemic activity. Administration of medicinal plants in fish can significantly reduce blood glucose and limit the hyperglycemic effects of environmental stressors or infections (Bulfon et al., 2015). Feed containing 4 ml/kg of cinnamon oil reduced glucose levels in *Oncorhynchus mykiss* (Kesbic, 2019). A decrease in blood glucose of *Oreochromis niloticus* fed 1% cinnamon was reported (Ali et al., 2020). TO used for 1 week at a dietary dose of 100 ml/kg reduced glucose values compared to control in *Oncorhynchus mykiss* (Gulec et al., 2013).

In this study, no significant effects of TC or TO on cellular composition of hematopoietic tissue of *Cyprinus carpio* juveniles were observed and in all the groups, hematopoietic tissue cellular structure was typical for healthy common carp (Kondera, 2014). No literature data concerning the hematopoietic effects of herbal feed supplements were found. Yilmaz and Ergun (2018) reported upregulation of interleukin-8 (IL-8), interleukin-1 (IL-1), transforming growth factor-α (TGF-α), and tumor necrosis factor-β (TNF-β) genes in the head kidney of *Oncorhynchus mykiss* fed 0.25–0.5% of cinnamic acid. According to Harikrishnan et al. (2021), dietary treatment with 5–15 mg/kg upregulated chemokine gene expression in head kidney leukocytes in the healthy and fungus-infected *Channa striata*. These data suggest the possible non-specific immunostimulatory effects of cinnamon products.

## Conclusion

The results of this study indicate no considerable hematotoxic effects of TC or TO used as therapeutic feed additives at the levels proved to be effective *in vitro* against various pathogenic *Aeromonas* spp. strains. Therefore, the attempts of the therapeutic use of these herbal compounds against fish pathogenic bacteria can be carefully undertaken. However, further studies including longer exposure to TC and TO are necessary to conclude about their safety.

## Data Availability Statement

The raw data supporting the conclusions of this article will be made available by the authors, without undue reservation.

## Ethics Statement

The animal study was reviewed and approved by I Local Ethics Committee in Warsaw, University of Warsaw, Poland.

## Author Contributions

BB, BK, and MW contributed to the conceptualization. EK, KŁ, BK, and MW contributed to the curation of data. EK and MW contributed to the funding acquisition and software. EK, BB, KŁ, and MW contributed to the investigation. EK, BB, BK, and MW contributed to the methodology, resources, and validation. MW contributed to the project administration. BB and MW contributed to the supervision. EK, BB, and MW contributed to formal analysis, writing, review, and editing of the original manuscript. All authors contributed to the article and approved the submitted version.

## Conflict of Interest

The authors declare that the research was conducted in the absence of any commercial or financial relationships that could be construed as a potential conflict of interest.

## Publisher’s Note

All claims expressed in this article are solely those of the authors and do not necessarily represent those of their affiliated organizations, or those of the publisher, the editors and the reviewers. Any product that may be evaluated in this article, or claim that may be made by its manufacturer, is not guaranteed or endorsed by the publisher.
